# Toxicological Assessment of the Marine-Derived Cyclic Dipeptide Cyclo(Pro-Tyr) Using In Vitro, Zebrafish Embryo, and BALB/c Mouse Models

**DOI:** 10.3390/jox16050170

**Published:** 2026-09-09

**Authors:** Shana Balachandran, Madan Kumar Arumugam, Rakhee Rathnam Kalari Kandy

**Affiliations:** 1 Cancer Biology Lab, Centre for Molecular and Nanomedical Sciences, Sathyabama Institute of Science and Technology, Chennai 600119, Tamil Nadu, India; 2 Department of Critical Care Medicine, University of Texas MD Anderson Cancer Center, Houston, TX 77030, USA

**Keywords:** Cyclo(Pro-Tyr), marine cyclic dipeptide, diketopiperazine, toxicological assessment, zebrafish embryo, sub-acute toxicity, Hepatotoxicity, repeated-dose toxicity

## Abstract

Cyclo(Pro-Tyr), a marine-derived cyclic dipeptide, has demonstrated anticancer activity, but its toxicological profile remains insufficiently characterized. The present study evaluated the preliminary toxicological effects of Cyclo(Pro-Tyr) using complementary in vitro cytotoxicity, zebrafish embryo developmental toxicity, and sub-acute oral toxicity in mice models. Cytotoxicity was assessed in 3T3-L1, HEK293, and HepG2 cells following 24 h exposure to Cyclo(Pro-Tyr) at 0–250 µM. Developmental toxicity was evaluated in zebrafish embryos exposed to 0–1000 µM for up to 96 h post-fertilization. A repeated-dose oral toxicity study was conducted in male and female BALB/c mice administered Cyclo(Pro-Tyr) at 100 or 500 mg/kg/day for 30 consecutive days, followed by hematological, serum biochemical, organ-weight, and histopathological assessments. Cyclo(Pro-Tyr) produced concentration-dependent reductions in cellular viability, with pronounced effects at the upper concentrations tested. In zebrafish embryos, exposure to 750 and 1000 µM was associated with increased mortality, reduced hatchability, developmental malformations, and reduced body length. In mice, repeated oral administration did not produce mortality, overt clinical toxicity, or significant changes in body weight or relative liver and kidney weights. However, dose-dependent alterations in selected hematological and serum biochemical parameters were observed, particularly at 500 mg/kg, accompanied by mild inflammatory and degenerative changes in hepatic and renal tissues. Overall, Cyclo(Pro-Tyr) exhibited concentration-dependent cellular and developmental toxicity at higher exposure concentrations and produced mild systemic alterations following repeated oral administration at the higher tested dose. These findings provide preliminary toxicological information relevant to the further preclinical evaluation of Cyclo(Pro-Tyr) as a marine-derived bioactive compound.

## 1. Introduction

Marine organisms represent a prolific source of structurally diverse bioactive metabolites with significant therapeutic potential, particularly in the field of anticancer drug discovery [1,2,3]. Among marine-derived compounds, cyclic dipeptides or 2,5-diketopiperazines (DKPs) have attracted considerable scientific attention due to their compact cyclic scaffold, metabolic stability, resistance to enzymatic degradation, and ability to interact with multiple biological targets [4,5]. Their rigid conformation and favorable physicochemical properties contribute to improved membrane permeability and pharmacological activity, making them promising candidates for translational drug development [6,7]. In recent years, several marine-derived DKPs have demonstrated antimicrobial, antioxidant, anti-inflammatory, and anticancer properties, further highlighting their biomedical relevance [8,9,10].

**Figure 1:**
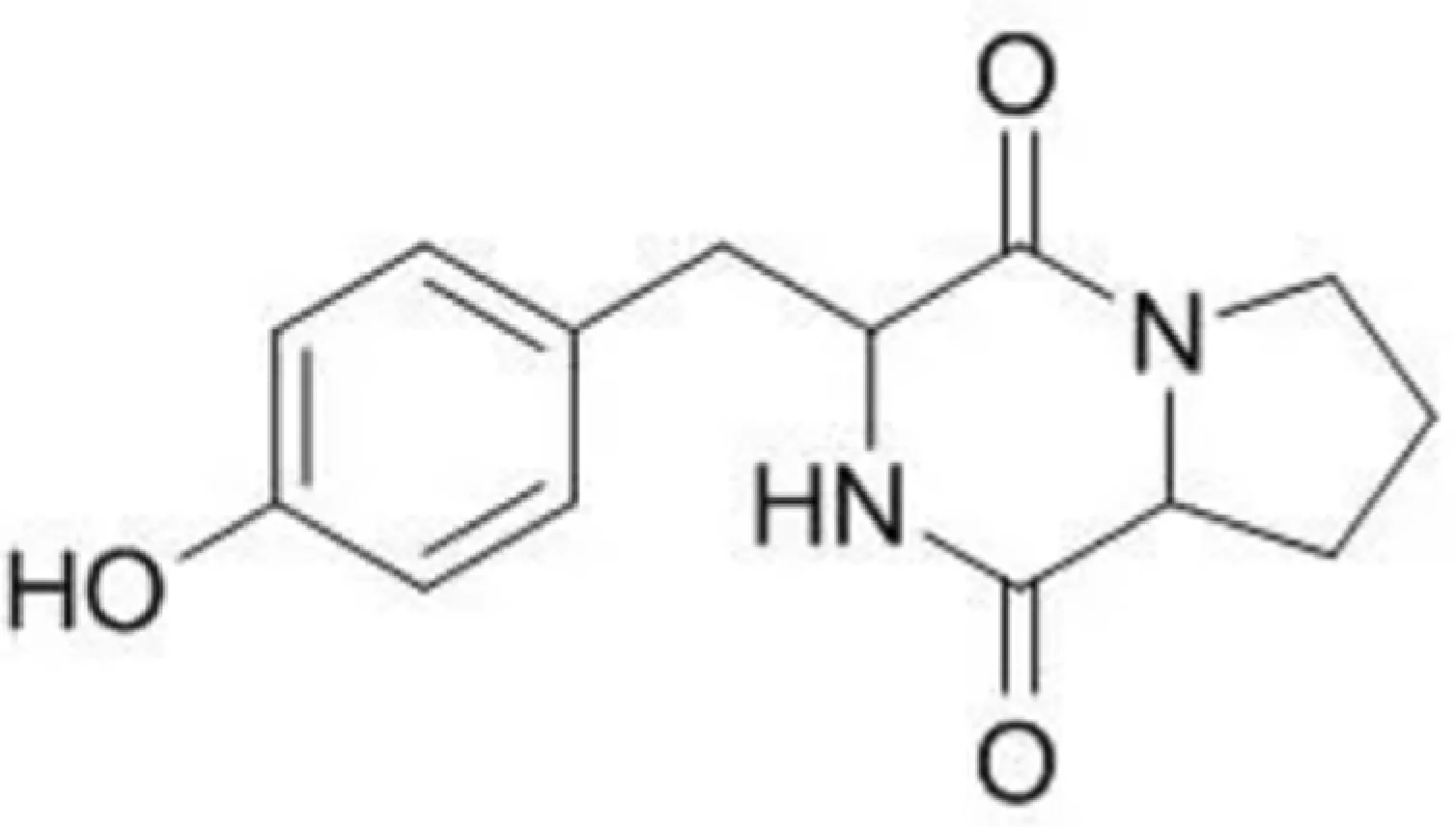
Structure of Cyclo(Pro-Tyr).

Cyclo(Pro-Tyr), also referred to as cyclo(L-Pro-L-Tyr) (Figure 1), is a proline-tyrosine diketopiperazine isolated from marine microbial sources. Cyclo(Pro-Tyr) was isolated and characterized in our laboratory from the marine sponge *Callyspongia fistularis*-associated symbiont *Bacillus pumilus* AMK1 [7,11]. Structurally related diketopiperazines have been reported to exhibit selective cytotoxicity against tumor cells through the induction of apoptosis, modulation of oxidative stress, and disruption of mitochondrial function [12,13] and Cyclo(Pro-Tyr) has shown anticancer activity in Hepatocellular carcinoma cells, inducing apoptosis, increased ROS generation, disrupted mitochondrial function, and exhibited no toxicity in normal fibroblast cells [7]. As above findings, suggest that proline-containing cyclic dipeptides may serve as valuable scaffolds for the development of targeted anticancer therapeutics. Previous investigations from our lab demonstrated that Cyclo(Pro-Tyr) induces apoptosis and mitochondrial dysfunction in hepatocellular carcinoma models through modulation of PI3K/AKT signaling [7,14]. Furthermore, our recent studies showed that Cyclo(Pro-Tyr) suppresses breast cancer cell proliferation by interfering with cyclin-dependent kinase signaling and cell cycle regulatory mechanisms [15,16]. Collectively, these findings establish Cyclo(Pro-Tyr) as a promising marine-derived anticancer lead with potential multitarget activity. Despite the growing evidence supporting the anticancer efficacy of Cyclo(Pro-Tyr) and related diketopiperazines, comprehensive toxicological evaluation remains largely unexplored. Therefore, systematic toxicological evaluation is essential before further translational development of Cyclo(Pro-Tyr). HepG2 cells were included as a hepatic metabolism-relevant model because they are widely used in studies of liver toxicity and cancer-related cytotoxicity [17,18].

Marine-derived anticancer peptides warrant comprehensive safety assessment because their bioactivity is frequently associated with membrane disruption and possible organ-specific toxicities [19] In line with a risk-based approach, peptides that remain stable and may enter systemic circulation should be evaluated for toxicity endpoints. Rapid *in vivo* toxicity models for peptides further demonstrate that potent anticancer activity can be accompanied by increased host toxicity, highlighting the importance of integrated preclinical safety testing [20]. Marine anticancer peptides are especially promising because many show strong tumor selectivity with relatively low toxicity to normal cells in preclinical models [19]. However, safety is not universal across this class, as some clinically studied marine peptides have been linked to dose-dependent toxicities, including elevated hepatic enzymes. Overall, although marine-derived anticancer peptides often display selective cytotoxicity against cancer cells, their translational potential depends on rigorous toxicity profiling, since both preclinical and clinical evidence show that normal-cell toxicity, organ safety, and *in vivo* tolerance are essential endpoints [21].

**Figure 2:**
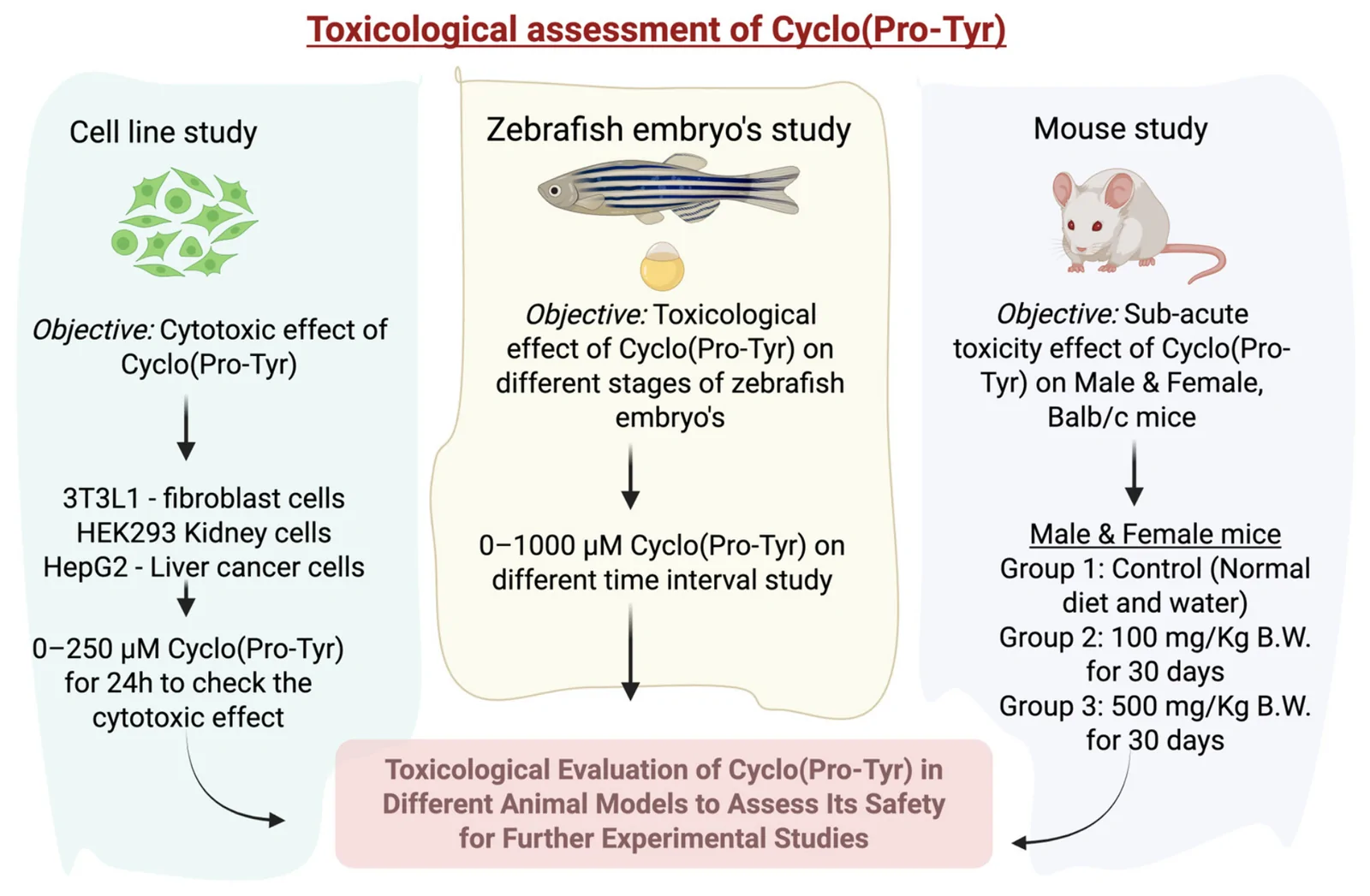
Schematic representation of experimental plan for the toxicological assessment of Cyclo(Pro-Tyr). Created in Biorender. Rakhee Rathnam Kalari Kandy. (2026) https://app.biorender.com/illustrations/64e7794b4f3ad93abd55a22f?slideId=9a9440a4-c909-4f01-a4d7-698991fef408.

A tiered toxicological approach integrating *in vitro*, zebrafish embryo developmental [20], and mammalian models provides a robust framework for preclinical safety assessment, as shown in Figure 2. Cell-based assays offer an initial evaluation of cytotoxicity in non-target tissues [22], while zebrafish embryos have emerged as an important vertebrate model for developmental toxicity because of their rapid embryogenesis, optical transparency, genetic similarity to humans, and suitability for high-throughput screening [23,24]. HepG2 cells were included as a hepatic metabolism-relevant model. The zebrafish embryo toxicity assay has been standardized under OECD Test Guideline 236 for evaluating acute developmental toxicity and teratogenic effects of investigational compounds. In addition, rodent toxicity studies remain indispensable for assessing systemic tolerability, hematological alterations, biochemical disturbances, and organ-specific pathological changes relevant to mammalian physiology in both male and female BALB/c mice [25]. In preclinical studies, the inclusion of both sexes of mouse models validates the comprehensive assessment of potential sex-dependent differences in systemic toxicity, hematological and biochemical alternations. Hence, the present study, the toxicological profile of Cyclo(Pro-Tyr) was systematically evaluated using complementary *in vitro*, zebrafish embryo, and murine models to establish its preliminary safety profile and support further preclinical development as a marine-derived anticancer molecule.

## 2. Materials and Methods

### 2.1. Chemicals and Reagents

Cyclo(Pro-Tyr) was isolated from the marine sponge *Callyspongia fistularis*-associated symbiont *Bacillus pumilus* AMK1, following our previously established isolation and characterization protocol [7,11,16]. A stock solution was prepared in 0.5% dimethyl sulfoxide (DMSO) and diluted in the appropriate culture medium before use, with the final DMSO concentration kept at or below 0.5% (*v*/*v*) in all experiments. Dulbecco’s Modified Eagle Medium (DMEM), fetal bovine serum (FBS), trypsin-EDTA, and penicillin-streptomycin were obtained from a commercial supplier (Gibco, Thermo Fisher Scientific, Waltham, MA, USA). 3-(4,5-dimethylthiazol-2-yl)-2,5-diphenyltetrazolium bromide (MTT) was purchased from a commercial supplier (Sigma-Aldrich, St. Louis, MA, USA). All other reagents used were of analytical grade.

### 2.2. Cell Lines and Culture

Mouse fibroblast (3T3-L1) (1337/2025-26/PN9), Human embryonic kidney-derived cells (HEK293) (575/2026-27/PN30), and human hepatoma cells (HepG2) (50/2025-26/PN21) cell lines were obtained from the National Centre for Cell Science (NCCS), Pune, India. Cells were maintained in DMEM supplemented with 10% (*v*/*v*) heat-inactivated FBS and 1% (*v*/*v*) penicillin-streptomycin, and were cultured in a humidified atmosphere of 5% CO_2_ at 37 °C. On reaching approximately 80% confluence, cells were subcultured into fresh medium for subsequent experiments. Cells were routinely monitored for morphology and confluency using an inverted phase-contrast microscope. Only exponentially growing cells with viability greater than 95% were used for all experiments.

### 2.3. Cytotoxicity Assay (MTT)

The cytotoxic effect of Cyclo(Pro-Tyr) was determined using the MTT assay following our previously established protocol [7,15,16]. Cells were seeded in 96-well plates at a density of 1 × 10^4^ cells per well and allowed to adhere. Following incubation, cells were treated with Cyclo(Pro-Tyr) at concentrations of 0, 50, 100, 150, 200, and 250 µM for 24 h. Control cells received vehicle alone containing 0.5% DMSO. After treatment, 20 µL of MTT reagent (5 mg/mL in phosphate-buffered saline) was added to each well and incubated for 3–4 h at 37 °C. The culture medium was then carefully removed, and the resulting purple formazan crystals were dissolved in DMSO. Absorbance was measured at 570 nm using a microplate reader (EnSpire, PerkinElmer, Shelton, CT, USA). Cell viability was expressed as the percentage relative to untreated control cells.

#### Morphological Assessment

Changes in cell morphology following Cyclo(Pro-Tyr) treatment were examined by phase-contrast microscopy [16]. Treated and control cells were observed and imaged using an inverted phase-contrast microscope at 20× magnification to document alterations in cell density, shape, and adherence. Morphological features associated with cytotoxicity, including cell shrinkage, membrane blebbing, cytoplasmic vacuolization, reduced cell density, loss of adherence, and rounding of cells, were qualitatively assessed and compared with untreated control cells [7].

### 2.4. Zebrafish Embryo Acute Toxicity Test

Developmental toxicity of Cyclo(Pro-Tyr) was evaluated using zebrafish (*Danio rerio*) embryos following the endpoints informed by Organisation for Economic Co-operation and Development (OECD) Test Guideline 236 for Fish Embryo Acute Toxicity (FET) testing [26]. Adult zebrafish were maintained under standard laboratory conditions with a 14 h light/10 h dark photoperiod at 28 ± 1 °C. Fertilized embryos were obtained by natural pairwise mating and collected within 2 h post-fertilisation (hpf) [27]. Viable embryos were selected under a stereomicroscope for further experimentation. Healthy fertilized embryos were distributed into multi-well plates, then exposed to Cyclo(Pro-Tyr) at concentrations of 0, 250, 500, 750, and 1000 µM in embryo medium. Cyclo(Pro-Tyr), stock solution was prepared at a concentration of 1 mM in 0.5% DMSO using E3 embryo medium. Approximately 10 embryos were placed in each well of a 24-well plate containing the respective treatment solution. Totally 30 embryos/group (10 embryos × 3 replicates). vehicle control containing equivalent DMSO concentration maintained in embryo medium served as the control group. The exposure period was carried out from 0 to 96 hpf under controlled laboratory conditions at 28 °C. Embryos were observed at 24, 48, 72, and 96 hpf using a stereomicroscope for morphological and physiological endpoints including mortality, hatchability, heart rate, malformation rate, and body length, and developmental abnormalities such as unformed head, unformed tail, pericardial edema, tail bending, spinal curvature, and reduced pigmentation were documented [27]. Mortality was determined based on coagulation of embryos, absence of somite formation, lack of tail detachment, and absence of heartbeat. Hatching rate was calculated as the percentage of embryos successfully hatched at each observation period. Body length measurements of hatched larvae were determined at 96 hpf using ImageJ analysis software (National Institute of Health (NIH), Bethesda, MD, USA). Heart rate was assessed by manually counting beats per minute under microscopic observation in randomly selected embryos from each treatment group.

### 2.5. Sub-Acute Repeated-Dose Toxicity Study in Mice

The sub-acute repeated-dose oral toxicity study was conducted in accordance with reference to the Organization for Economic Co-operation and Development (OECD) Test Guideline for repeated-dose oral toxicity studies in rodents [28,29], with the exposure period to 30 days. All animal experimental procedures were carried out with the institutional ethical approval IAEC# SU/CLATR/IAEC/XXVI/82/2025. Healthy adult male and female BALB/c mice weighing approximately 20–25 g were procured from an approved laboratory animal facility and acclimatized for one week prior to experimentation. Animals were maintained under standard laboratory conditions at 22 ± 2 °C with a relative humidity of 50–60% under a 12 h light/dark cycle. Standard pellet diet and water were provided ad libitum throughout the study period [30]. Animals were randomly allocated into three groups of six animals each per sex *(n* = 6 per group). Group 1 served as the untreated/vehicle control and received a normal diet and water. Group 2 and Group 3 received Cyclo(Pro-Tyr) at 100 and 500 mg/kg body weight, respectively. Cyclo(Pro-Tyr) was prepared in 0.5% DMSO using phosphate-buffered saline (PBS) and administered by oral gavage at a dosing volume of once daily for 30 consecutive days. Animals were observed daily for clinical signs of toxicity, morbidity, and mortality throughout the experimental period. Animals were observed for clinical signs throughout the study, and body weights were recorded on day 0, day 14, and day 30 using a digital weighing balance. At the end of the study period, animals were euthanized using ketamine (70 mg/kg, i.p.) and xylazine (10 mg/kg, i.p.) [31], blood samples were collected for hematological and serum biochemical analyses.

#### 2.5.1. Hematology and Serum Biochemistry

For hematological evaluation, whole blood samples were collected in EDTA-coated tubes and analyzed for parameters including red blood cell count (RBC), white blood cell count (WBC), hemoglobin concentration (Hb), platelet count, and differential leukocyte count using an automated hematology analyzer (Erba H360 hematology analyzer, Transasia Bio-Medicals Ltd., Mumbai, India) [32]. For serum biochemical analysis, blood samples were centrifuged at 3000 rpm for 10 min to obtain serum. Liver function markers including alanine aminotransferase (ALT), aspartate aminotransferase (AST), alkaline phosphatase (ALP), and total bilirubin were assessed. Renal function parameters including urea and creatinine were also analyzed using standard diagnostic kits according to the manufacturer’s instructions.

#### 2.5.2. Body Weight and Organ Weight

Individual body weights were recorded on day 0, day 14, and day 30 of the study. Following necropsy, liver and kidney tissues were carefully excised, rinsed with ice-cold saline, blotted dry, and weighed. Absolute organ weights were recorded for male and female animals in each group. Relative organ weights were calculated based on body weight.

Relative organ weight (%) = (organ weight/body weight) × 100

#### 2.5.3. Histopathology

For histopathological evaluation, liver and kidney tissues from control and treated animals were fixed, processed, embedded, sectioned, and stained with hematoxylin and eosin (H&E) [14]. Tissue samples were fixed in 10% neutral buffered formalin and fixed tissues were processed using standard paraffin embedding techniques, sectioned at approximately 4–5 µm thickness, and stained with H&E. Histological alterations in liver and kidney architecture were examined under a light microscope at 10× magnification with selected regions viewed at higher magnification to assess tissue architecture and any treatment-related changes and documented photographically.

### 2.6. Statistical Analysis

Results from the cell line and zebrafish experiments are expressed as mean ± standard error of the mean (SEM) based on three independent experiments (*n* = 3), while results from the mouse study are expressed as mean ± standard deviation (SD) for six animals per group (*n* = 6). Data were analyzed by one-way analysis of variance (ANOVA) followed by Tukey’s post hoc test for intergroup comparisons using GraphPad Prism software Prism 8.0.2 (Dotmatics, MA, USA). Differences were considered statistically significant at *p* ≤ 0.05.

## 3. Results

### 3.1. Cytotoxicity Effect of Cyclo(Pro-Tyr)

**Figure 3:**
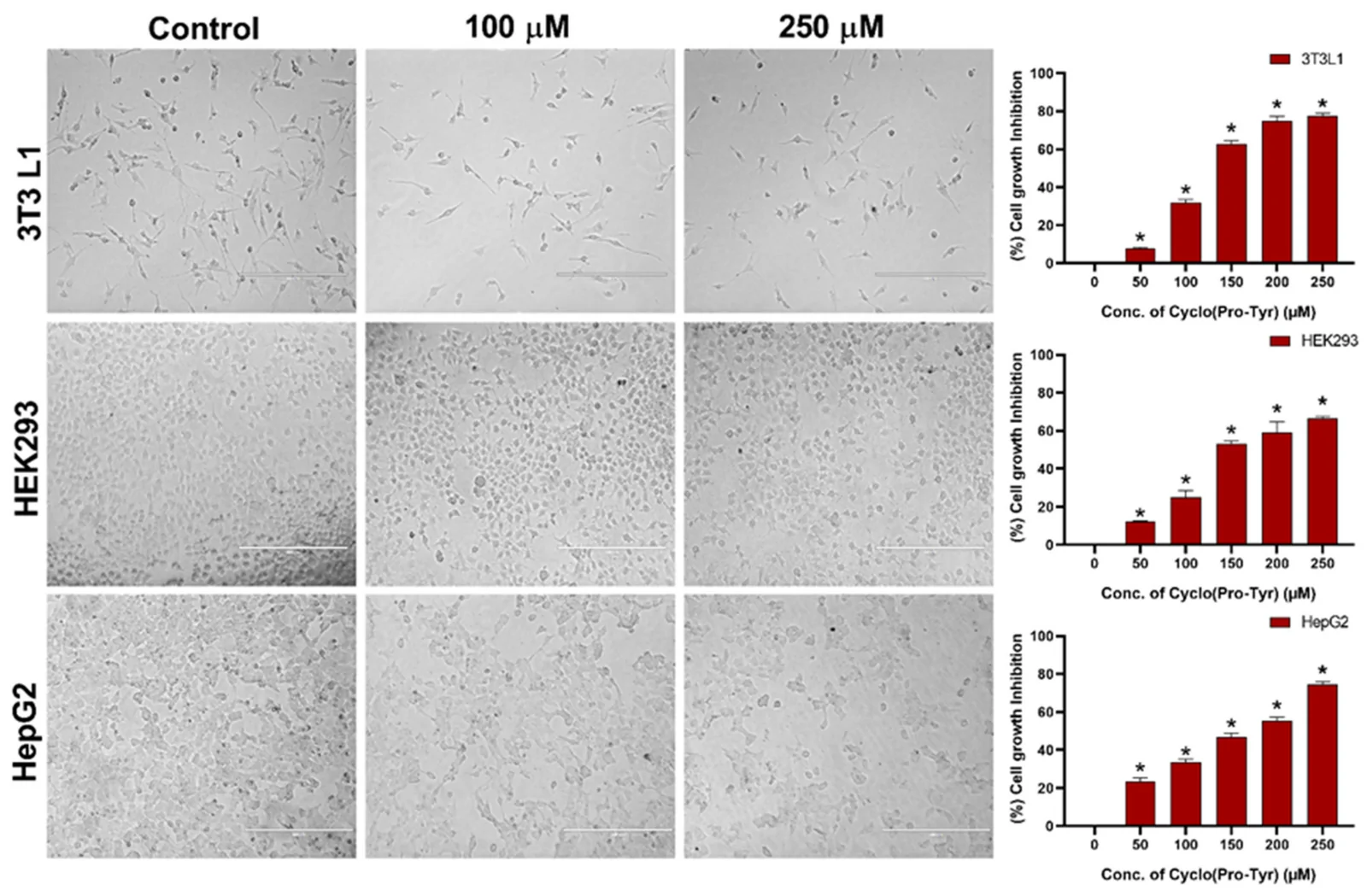
Cytotoxicity assessment of Cyclo(Pro-Tyr) by morphological changes and MTT assay in fibroblast (3T3L1) cells, Human embryonic kidney-derived cells (HEK293) and HepG2 liver cells. Cells were exposed to different concentrations (0–250 µM) of Cyclo(Pro-Tyr) for 24 h. Morphological images were grabbed using a inverted phase contrast microscope at 20× magnifications (Scale 100 µm). (*n* = 3), Values represent significant at * *p* < 0.05 vs. Control. (The unprocessed original image can be found in the Supplementary Materials.)

The cytotoxic effect of Cyclo(Pro-Tyr) was assessed in 3T3-L1 fibroblast, Human embryonic kidney-derived HEK293 cells and HepG2 cells exposed to increasing concentrations (0–250 µM) for 24 h, using the MTT assay together with phase-contrast morphology (Figure 3). In all three cell lines, Cyclo(Pro-Tyr) produced a clear concentration-dependent increase in cell growth inhibition. In 3T3-L1 cells, inhibition rose from approximately 8% at 50 µM to approximately 77% at 250 µM. In HEK293 cells, inhibition increased from approximately 12% at 50 µM to approximately 67% at 250 µM. In HepG2 cells, inhibition rose from approximately 23% at 50 µM to approximately 75% at 250 µM Morphological assessment using inverted phase-contrast microscopy further supported the cytotoxic findings. Untreated control cells maintained normal morphology with intact membrane structure, characteristic spindle-shaped or polygonal appearance, and dense monolayer growth. In contrast, Cyclo(Pro-Tyr)-treated cells displayed concentration-dependent morphological alterations including reduction in cell density, loss of adherence, cell shrinkage, rounding of cells, cytoplasmic condensation, and membrane irregularities. These changes became more prominent at higher concentrations, particularly at 200 and 250 µM, indicating progressive cellular stress and cytotoxic damage following Cyclo(Pro-Tyr) exposure (Figure 3). These results indicate that Cyclo(Pro-Tyr) exerts a dose-dependent cytotoxic effect across cells of fibroblast, renal, and hepatic origin, with the most pronounced effects occurring at the upper end of the tested range. Among the tested cell lines, HepG2 cells exhibited comparatively greater sensitivity to Cyclo(Pro-Tyr) exposure, whereas 3T3-L1 and HEK293 cells demonstrated moderate reductions in viability across the concentration range tested, when compared to control groups.

### 3.2. Cyclo(Pro-Tyr) Causes Concentration- and Time-Dependent Developmental Toxicity in Zebrafish Embryos

**Figure 4:**
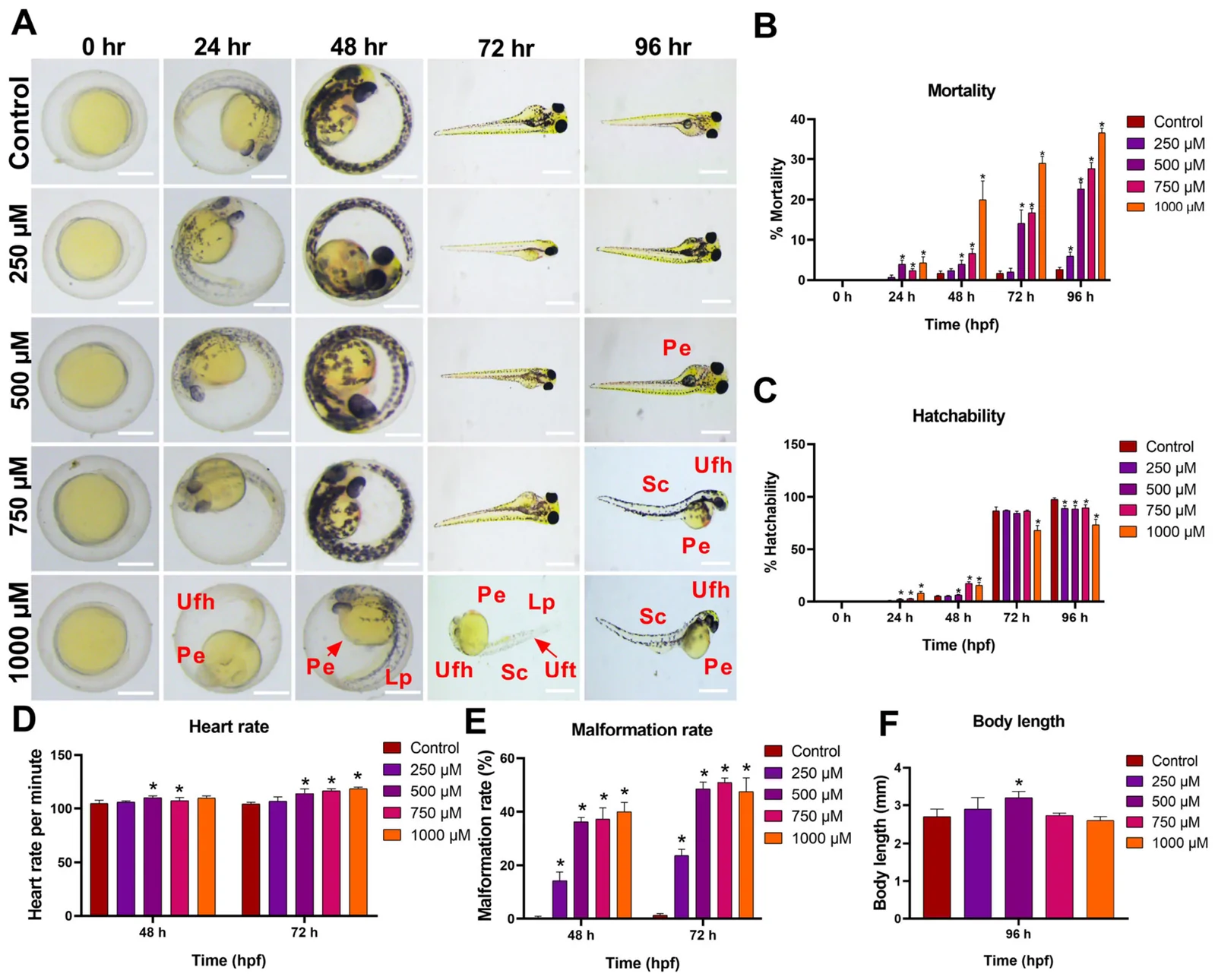
Microscope images of zebrafish embryos exposed to different concentrations of Cyclo(Pro-Tyr) for different time intervals, displaying lethal and sub-lethal effects, compared to the negative controls. (**A**) These effects were imaged at each stage of the normal development process of embryos in the control and experimental groups. Typical pictures of each stage of the development process of embryos at various concentrations (250, 500, 750 and 1000 μM). Ufh-Unformed head; Uft-Unformed tail; Pe-Pericardial edema; Tb-Tail bent; Sc- Spinal curvature; Lp-less pigmented. Higher concentration shows a sign of growth retardation and no pigmentation, weak or no heart beat and blood circulation. (**B**) % mortality (**C**) % Hatchability, (**D**) Heart rate (beats/min), (**E**) % malformation rate and (**F**) Body length in mm were noted at different concentrations of Cyclo(Pro-Tyr) in different time intervals. (*n* = 3), Values are expressed as mean ± SD (*n* = 3). * *p* < 0.05 vs. Control. (The unprocessed original image can be found in the Supplementary Materials).

To evaluate developmental toxicity, zebrafish embryos were exposed to Cyclo(Pro-Tyr) at concentrations of 0, 250, 500, 750, and 1000 µM and monitored from 0 to 96 h post-fertilization (hpf) (Figure 4). Cyclo(Pro-Tyr) exposure produced concentration- and time-dependent lethal and sub-lethal effects throughout embryonic development. Embryonic mortality remained minimal in the control group but progressively increased with increasing Cyclo(Pro-Tyr) concentration and exposure duration. Mortality was low at early time points and at lower concentrations but rose progressively, reaching approximately 36% at 1000 µM by 96 hpf (Figure 4B). Higher concentrations, produced marked increases in mortality by 96 hpf, with the highest concentration exhibiting the greatest embryonic lethality (Figure 4B). In contrast, embryos exposed to lower concentrations showed comparatively reduced mortality compared to control groups.

Hatchability analysis demonstrated that lower Cyclo(Pro-Tyr) concentrations were relatively well tolerated, whereas embryos exposed to higher concentrations exhibited delayed or reduced hatching rates. The reduction in hatchability became more evident at 750 and 1000 µM during later developmental stages (Figure 4C). Hatchability was largely preserved at lower concentrations but declined at the highest concentrations, falling to approximately 70% at 1000 µM by 96 hpf, compared to control. Heart rate remained detectable throughout exposure, although embryos exposed to 750 and 1000 µM exhibited a modest reduction compared with controls and remained within a narrow range across groups, in the order of 105 to 118 beats per minute (Figure 4D). Although embryos exposed to higher Cyclo(Pro-Tyr) concentrations showed a slight reduction in beats per minute compared with controls, cardiac activity remained detectable throughout the exposure period (Figure 4D). Developmental malformations increased markedly in a concentration-dependent manner, particularly at 48 and 72 hpf and higher Cyclo(Pro-Tyr) concentrations (Figure 4E). Morphological abnormalities observed included unformed head and tail structures, pericardial edema, yolk sac edema, tail bending, spinal curvature, reduced pigmentation, developmental delay, and impaired circulation. Embryos exposed to 750 and 1000 µM additionally showed signs of growth retardation, weak heartbeat, and reduced blood circulation (Figure 4A). The malformation rate increased markedly with concentration and time, reaching approximately 48 to 51% at 72 h in the higher concentration groups (Figure 4E), and body length at 96 h ranged from approximately 2.6 to 3.2 mm across groups (Figure 4F). The reduction was statistically significant at 750 and 1000 µM (*p* < 0.05).

Embryos treated with higher concentrations exhibited visibly reduced body length compared with control larvae, indicating concentration-dependent growth inhibition (Figure 4F). Morphological examination confirmed a range of developmental defects at 750 and 1000 µM, including unformed head and tail, pericardial edema, tail bending, spinal curvature, and reduced pigmentation, together with signs of growth retardation and weak or absent heartbeat and circulation at the highest concentration (Figure 4A). Lower concentrations were comparatively well tolerated, indicating a concentration-dependent developmental sensitivity to Cyclo(Pro-Tyr). Collectively, these findings demonstrate that Cyclo(Pro-Tyr) induces concentration- and time-dependent developmental toxicity in zebrafish embryos, with severe teratogenic and lethal effects predominantly observed at higher exposure concentrations, compared to control.

### 3.3. Sub-Acute Repeated-Dose Toxicity of Cyclo(Pro-Tyr) in BALB/c Mice

**Figure 5:**
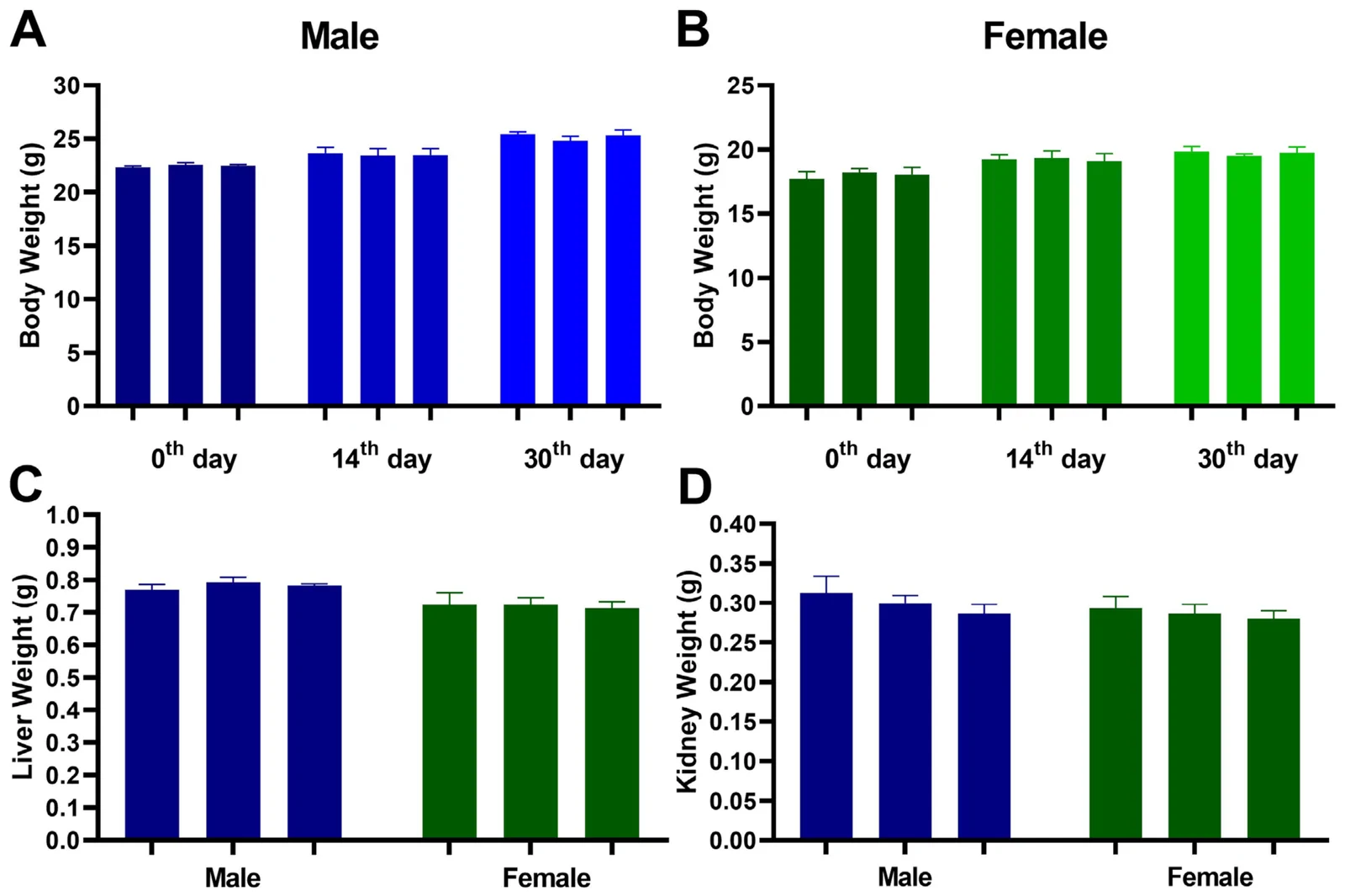
Effect of Cyclo(Pro-Tyr) on (**A**) male mice body weight, (**B**) female mice body weight, (**C**) male and female mice liver weight and (**D**) male and female mice kidney weight index in mice of control (group 1), 100 mg/kg body weight of Cyclo(Pro-Tyr) (group 2) and 500 mg/kg body weight of Cyclo(Pro-Tyr) (group 3). The data were expressed as mean ± SD (*n* = 6).

**Figure 6:**
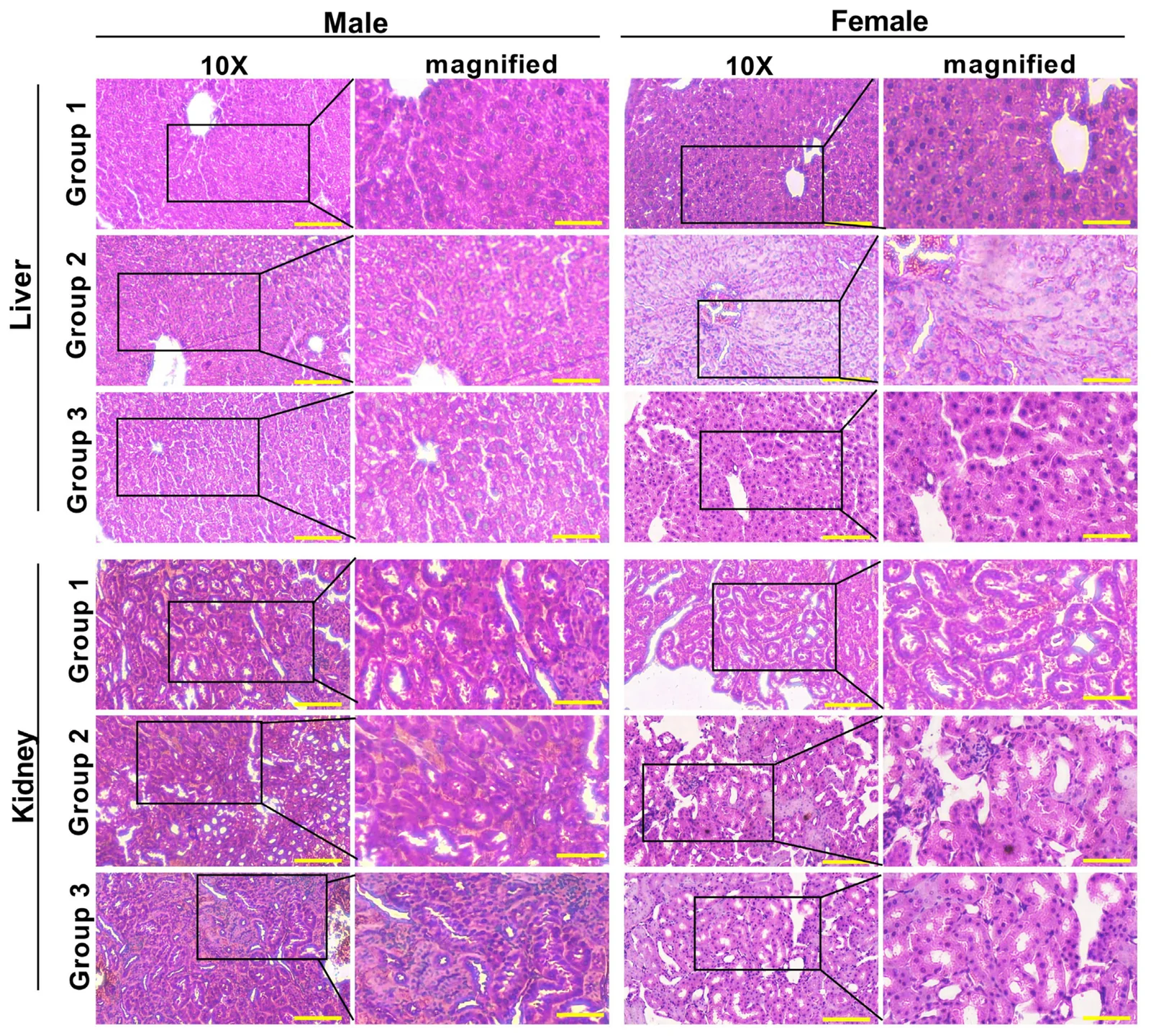
Histochemical analysis and localization by H&E staining of the liver and kidney of mice administrated with control (group 1), 100 mg/kg body weight of Cyclo(Pro-Tyr) (group 2), and 500 mg/kg body weight of Cyclo(Pro-Tyr) (group 3) experimental animals. Histology sections of liver from Group 1 of male and female mice showing normal cellular structure. Section of the liver from Group 2 and group 3 mice reveals liver perivascular cell infiltration and few necrosis in the hepatocytes. Section of kidney from Group 1 male and female mice showing, normal histologic structure. Section of kidney from Group 2 and 3 a small number of infiltrations. (The unprocessed original image can be found in the Supplementary Materials.)

The sub-acute repeated-dose oral toxicity of Cyclo(Pro-Tyr) was evaluated in male and female BALB/c mice administered Cyclo(Pro-Tyr) at 100 and 500 mg/kg body weight for 30 consecutive days (Figure 5 and Figure 6). Throughout the experimental period, no mortality or severe clinical signs of toxicity were observed in any treatment group.

#### Cyclo(Pro-Tyr) Does Not Significantly Alter Body Weight or Relative Organ Weights in Mice

In the 30-day sub-acute study, body weight and organ weight were monitored in male and female mice treated with Cyclo(Pro-Tyr) at 100 or 500 mg/kg and compared with untreated controls (Figure 5). Body weight increased steadily over the study period in all groups, with comparable values recorded on day 0, day 14, and day 30 in both sexes, and no significant difference between treated and control animals (Figure 5A,B). Relative liver and kidney weights were also comparable across the control and treated groups in both male and female animals, with no statistically significant treatment-related change (Figure 5C,D). These observations indicate that repeated oral administration of Cyclo(Pro-Tyr) at 100 and 500 mg/kg for 30 days did not adversely affect growth or gross organ mass under the conditions tested.

### 3.4. Effect of Cyclo(Pro-Tyr) on Hematological Parameters

Hematological parameters were assessed at the end of the 30-day study to evaluate systemic and organ-specific effects of Cyclo(Pro-Tyr). The effects of repeated oral administration of Cyclo(Pro-Tyr) on hematological parameters in male and female BALB/c mice are presented in Table 1. No marked alterations were observed in erythrocytic indices including red blood cell count, hemoglobin concentration, hematocrit, mean corpuscular volume, mean corpuscular hemoglobin, and mean corpuscular hemoglobin concentration following 30 days of treatment. These findings suggest that Cyclo(Pro-Tyr) did not induce anemia or adversely affect erythropoiesis at the tested doses. White blood cell counts exhibited a mild dose-dependent increase in both sexes, with the highest values observed in the 500 mg/kg treatment group. Differential leukocyte analysis revealed a moderate increase in neutrophil and monocyte percentages accompanied by a slight reduction in lymphocyte percentages at the higher dose. Platelet counts showed a mild reduction at 500 mg/kg but remained within physiological reference ranges. Overall, the hematological profile indicates that repeated Cyclo(Pro-Tyr) administration did not produce significant haematotoxin effects, although mild leukocytic changes were observed at the higher dose, consistent with a low-grade inflammatory response.

### 3.5. Effect of Cyclo(Pro-Tyr) on Serum Biochemical Parameters

Serum biochemical parameters evaluated after 30 days of repeated oral Cyclo(Pro-Tyr) administration are summarized in Table 2. Liver function markers demonstrated a dose-dependent increase in both male and female mice. Serum AST, ALT, and ALP activities were moderately elevated in the 500 mg/kg group compared with controls, whereas total bilirubin levels remained largely unchanged. The maintenance of normal bilirubin concentrations together with only moderate elevations in hepatic enzymes suggests mild hepatocellular stress rather than severe hepatic dysfunction.

Total protein and albumin concentrations showed only minor reductions across treatment groups and remained within physiological limits, indicating preservation of hepatic synthetic function. Serum glucose and total cholesterol levels exhibited modest increases at the higher dose but did not indicate marked metabolic disturbance Renal function assessment revealed mild dose-dependent increases in serum urea, creatinine, and uric acid concentrations. However, these changes were relatively small and remained consistent with the preserved renal architecture observed histologically. Collectively, the biochemical findings indicate that repeated oral exposure to Cyclo(Pro-Tyr) induced mild hepatic and renal alterations at higher doses without evidence of severe structural organ injury under the conditions tested, supporting the conclusion that Cyclo(Pro-Tyr) was generally well tolerated under the experimental conditions employed.

### 3.6. Histopathological Evaluation of Liver and Kidney

Histopathological examination of liver and kidney tissues was performed following 30 days of repeated oral administration of Cyclo(Pro-Tyr) in male and female BALB/c mice (Figure 6). Representative H&E stained sections revealed generally preserved tissue architecture in both organs across all experimental groups. In the liver, sections obtained from control animals (Group 1) exhibited normal hepatic histology characterized by well-organized hepatic cords radiating from the central vein, intact sinusoidal spaces, and normal hepatocyte morphology without evidence of degeneration or inflammatory changes. In Cyclo(Pro-Tyr)-treated animals, the overall hepatic architecture remained largely preserved. Mice receiving 100 mg/kg Cyclo(Pro-Tyr) (Group 2) showed mild focal inflammatory cell infiltration within the periportal and perivascular regions. Similar findings were observed in the 500 mg/kg group (Group 3), where mild inflammatory infiltration was accompanied by occasional focal hepatocellular degeneration and isolated necrotic hepatocytes. However, extensive hepatocellular necrosis or severe disruption of hepatic architecture were not observed in either sex. Histological evaluation of kidney sections from control animals demonstrated normal renal morphology, including intact glomeruli, well-defined Bowman’s capsules, and preserved proximal and distal renal tubules. Cyclo(Pro-Tyr)-treated groups exhibited largely normal renal architecture with only minor histopathological alterations. Animals administered 100 mg/kg Cyclo(Pro-Tyr) displayed occasional focal inflammatory cell infiltration within the renal interstitial. Similar mild inflammatory changes were observed in the 500 mg/kg group, with preservation of glomerular and tubular structures. No evidence of glomerular degeneration, tubular necrosis, extensive inflammatory lesions, vascular damage, or severe nephropathological alterations was detected.

No appreciable sex-dependent differences in histopathological response were observed between male and female mice. The histological findings correlate with the modest elevations observed in serum hepatic and renal biomarkers and indicate that repeated oral administration of Cyclo(Pro-Tyr) for 30 days induced only mild tissue alterations at the tested dose levels. Overall, the preservation of normal hepatic and renal architecture supports the conclusion that Cyclo(Pro-Tyr) possesses a relatively favorable safety profile under the experimental conditions employed.

## 4. Discussion

The therapeutic value of drug candidate molecule depends, careful balance between therapeutic efficacy and systemic safety [33]. Although, Cyclo(Pro-Tyr) has previously demonstrated promising anticancer activity through induction of apoptosis [15,16], disruption of mitochondrial function [7], and modulation of cell-cycle regulatory pathways, information regarding its toxicological profile has remained limited. The present study therefore employed a tiered toxicological assessment strategy encompassing *in vitro* cellular models, zebrafish embryo developmental toxicity testing, and a sub-acute rodent toxicity study to establish a preliminary safety profile for Cyclo(Pro-Tyr) and identify potential target organs of toxicity. At the cellular level, Cyclo(Pro-Tyr) produced a concentration-dependent reduction in viability in fibroblast, kidney, and liver cancer cells, with substantial inhibition emerging mainly at the higher concentrations of the tested range. It is useful to view these results alongside the anticancer potency previously reported for Cyclo(Pro-Tyr), where a half-maximal inhibitory concentration of approximately 84.55 µM was observed in MCF-7 breast cancer cells [15,16]. The observation that marked cytotoxicity in the non-cancerous fibroblast and kidney derived cell lines appears largely at concentrations above this range is consistent, suggests a degree of selectivity toward cancer cells; however, formal assessment of the therapeutic window will require additional comparative pharmacological and toxicological studies.

In the zebrafish embryo model, Cyclo(Pro-Tyr) was comparatively well tolerated at lower concentrations, while the highest concentrations of 750 and 1000 µM produced increased mortality, reduced hatchability, a range of developmental malformations, and reduced body length. The clear dependence of these effects on both concentration and exposure time is typical of developmental toxicity and supports the use of the zebrafish embryo as an efficient bridge between cell-based assays and mammalian testing [34,35,36]. The occurrence of morphological abnormalities at the higher concentrations indicates that developmental toxicity should be considered when defining exposure levels for subsequent studies [27,37,38,39]. The repeated-dose murine study provided complementary information on systemic responses following oral administration. Repeated oral administration of Cyclo(Pro-Tyr) at 100 and 500 mg/kg for 30 days did not impair growth and was generally well tolerated in male and female BALB/c mice. No mortality, overt clinical signs of toxicity, or treatment-related behavioral abnormalities were observed throughout the study period. Furthermore, body weight gain occurred normally in all treatment groups, indicating that Cyclo(Pro-Tyr) did not significantly impair food utilization, metabolic homeostasis, or general physiological status [40]. Changes in body weight are widely regarded as sensitive indicators of systemic toxicity, and the absence of treatment-related weight loss suggests a relatively low level of whole-body toxicity [40,41,42].

Similarly, relative liver and kidney weights remained comparable to those of control animals. Organ weight alterations frequently represent early indicators of toxicant-induced organ dysfunction before the appearance of overt histopathological lesions. Therefore, the lack of significant changes in organ weight supports the conclusion that Cyclo(Pro-Tyr) did not induce substantial organ hypertrophy, atrophy, or pathological enlargement during the exposure concentrations and period [40,42,43]. Hematological evaluation revealed minimal effects on erythrocytic parameters including red blood cell count, hemoglobin concentration, hematocrit, MCV, MCH, and MCHC, indicating that Cyclo(Pro-Tyr) did not adversely affect erythropoiesis or induce treatment-related anemia. Platelet counts also remained within normal physiological limits, suggesting preserved hematopoietic function [44]. Mild elevations in total white blood cell counts together with increased neutrophil and monocyte percentages were observed at the higher dose level [45,46]. The concurrent increase in leukocyte-associated parameters and the mild inflammatory infiltrates observed histologically may be consistent with a low-grade inflammatory response; however, this interpretation would require confirmation using inflammatory biomarkers [47,48]. Biochemical analysis identified mild dose-dependent increases in AST, ALT, and ALP activities in both sexes [49]. Elevations in these enzymes are commonly interpreted as indicators of hepatocellular membrane perturbation and leakage of intracellular enzymes into circulation [50]. Thus, supports the histological examination of hepatic and renal architecture, with only minor perivascular infiltration and occasional hepatocyte necrosis at the treated doses [51,52]. Taken together, these findings point to reasonable tolerability of Cyclo(Pro-Tyr) at the doses examined over a sub-acute exposure period, and they define a practical dose range for subsequent studies. Male and female mice exhibited comparable trends in body weight, organ weight, hematology, serum biochemistry, and histopathology [42,53]. Such consistency strengthens the reliability of the observed toxicological profile and suggests that No marked sex-related differences were apparent in the measured toxicological endpoints under the present experimental conditions; however, dedicated studies with sufficient statistical power would be required to establish whether Cyclo(Pro-Tyr) toxicity is modified by sex. Also, the determination of a formal no-observed-adverse-effect level will require larger dose-ranging studies incorporating additional dose levels and extended observation periods. Further work incorporating a broader dose range with longer exposure durations mechanistic endpoints will be studied.

## 5. Conclusions

This study provides a preliminary, multi-tier toxicological assessment of the marine-derived cyclic dipeptide Cyclo(Pro-Tyr) across cellular, developmental, and mammalian models. Cyclo(Pro-Tyr) showed concentration and exposure dependent toxicity that was most pronounced at the highest concentrations in the zebrafish embryo model, while repeated oral dosing at 100 and 500 mg/kg for 30 days was reasonably tolerated in mice, with no significant effect on growth or organ weights and only minor histological changes. The mild biochemical and histopathological alterations observed at 500 mg/kg indicate limited hepatic and renal involvement without substantial impairment of organ function. These results define a working preliminary tolerability range and provide a safety framework to guide the continued preclinical development of Cyclo(Pro-Tyr), and further mechanistic and longer-term studies.

## Figures and Tables

**Table 1:** Haematological parameters in male and female BALB/c mice after 30 days of Cyclo(Pro-Tyr) administration (mean ± SD, *n* = 6).

Hematological Parameter (Unit)	Male			Female		
	Group 1 (Control)	Group 2 (100 mg/kg)	Group 3 (500 mg/kg)	Group 1 (Control)	Group 2 (100 mg/kg)	Group 3 (500 mg/kg)
Red blood cells (×10^6^/µL)	8.10 ± 0.32	8.02 ± 0.41	7.85 ± 0.38	7.92 ± 0.28	7.85 ± 0.34	7.70 ± 0.39
Hemoglobin (g/dL)	14.2 ± 0.6	14.0 ± 0.5	13.7 ± 0.7	13.8 ± 0.5	13.7 ± 0.6	13.4 ± 0.6
Hematocrit (%)	42.8 ± 1.9	42.1 ± 2.1	40.9 ± 2.3 *	41.6 ± 1.8	41.0 ± 2.0	40.1 ± 2.1
Mean corpuscular volume (fL)	52.8 ± 2.1	52.5 ± 2.3	52.1 ± 2.4	52.5 ± 2.0	52.1 ± 2.2	51.8 ± 2.3
Mean corpuscular hemoglobin (pg)	17.5 ± 0.8	17.4 ± 0.9	17.2 ± 0.8	17.4 ± 0.7	17.3 ± 0.8	17.1 ± 0.9
MCH concentration (g/dL)	33.2 ± 1.1	33.0 ± 1.2	32.8 ± 1.3	33.1 ± 1.0	32.9 ± 1.1	32.7 ± 1.2
White blood cells (×10^3^/µL)	6.8 ± 0.7	7.1 ± 0.8 *	8.0 ± 0.9 *	6.4 ± 0.8	6.9 ± 0.7	7.8 ± 0.8 *
Neutrophils (%)	21.5 ± 2.8	23.2 ± 3.1	28.4 ± 3.5	20.8 ± 2.6	22.5 ± 2.8	27.1 ± 3.3 *
Lymphocytes (%)	73.1 ± 3.5	71.6 ± 3.7	67.2 ± 4.0 *	74.2 ± 3.2	72.9 ± 3.4	68.4 ± 3.8 *
Monocytes (%)	3.8 ± 0.6	4.2 ± 0.7	5.8 ± 0.8 *	3.6 ± 0.5	4.0 ± 0.6 *	5.5 ± 0.7 *
Platelets (×10^3^/µL)	790 ± 55	775 ± 62	750 ± 58 *	770 ± 48	760 ± 52	742 ± 55 *

Data are expressed as mean ± SD Statistical analysis was performed using one-way ANOVA followed by Tukey’s multiple comparison test. Differences were considered statistically significant at * *p* < 0.05.

**Table 2:** Serum biochemical parameters in male and female BALB/c mice after 30 days of Cyclo(Pro-Tyr) administration.

Biochemical Parameter (Unit)	Male			Female		
	Group 1 (Control)	Group 2 (100 mg/kg)	Group 3 (500 mg/kg)	Group 1 (Control)	Group 2 (100 mg/kg)	Group 3 (500 mg/kg)
Aspartate aminotransferase (IU/L)	92.3 ± 7.3	99.2 ± 8.5 *	115.0 ± 9.8 *	88.9 ± 8.3	96.1 ± 8.3 *	113.2 ± 9.5 *
Alanine aminotransferase (IU/L)	42.4 ± 3.5	47.9 ± 4.4 *	58.7 ± 5.3 *	41.4 ± 3.5	45.9 ± 4.4 *	56.7 ± 5.2 *
ALP (U/L)	108.5 ± 10.8	114.9 ± 11.2 *	126.7 ± 12.1 *	104.3 ± 8.6	110.1 ± 9.8 *	120.2 ± 10.8 *
Total bilirubin (mg/dL)	0.38 ± 0.03	0.41 ± 0.06	0.43 ± 0.09	0.36 ± 0.05	0.39 ± 0.07	0.41 ± 0.09
Total protein (g/dL)	6.8 ± 0.4	6.7 ± 0.5	6.5 ± 0.5	6.9 ± 0.4	6.8 ± 0.4	6.6 ± 0.5
Albumin (g/dL)	3.9 ± 0.2	3.8 ± 0.2	3.7 ± 0.3	4.0 ± 0.2	3.9 ± 0.2	3.8 ± 0.2
Urea (mg/dL)	28.4 ± 2.6	30.7 ± 2.8 *	34.7 ± 3.1 *	26.2 ± 2.6	31.7 ± 2.8 *	33.5 ± 3.1 *
Creatinine (mg/dL)	0.40 ± 0.05	0.45 ± 0.05	0.50 ± 0.06	0.40 ± 0.05	0.43 ± 0.05	0.48 ± 0.06
Uric acid (mg/dL)	1.8 ± 0.2	1.9 ± 0.2	2.1 ± 0.3	1.7 ± 0.2	1.8 ± 0.2	2.0 ± 0.3
Glucose (mg/dL)	108 ± 9	112 ± 10 *	118 ± 11 *	104 ± 8	109 ± 9	115 ± 10
Total cholesterol (mg/dL)	107.4 ± 10.8	115.2 ± 11.2 *	122.0 ± 12.6 *	109.0 ± 10.9	119.9 ± 11.5 *	126.7 ± 12.9 *

Values are expressed as mean ± SD (*n* = 6). Statistical significance was determined using one-way ANOVA followed by Tukey’s post hoc test. * *p* < 0.05 compared with the respective control group.

## Data Availability

The original contributions presented in this study are included in the article. Further inquiries can be directed to the corresponding authors.

## Supplementary Materials

The following supporting information can be downloaded at: https://www.mdpi.com/article/10.3390/jox16050170/s1. The unprocessed original images of Figure 3, Figure 4 and Figure 6 can be found in the Supplementary Materials.
